# Elaboration of Gluten-Free Cookies with Defatted Seed Flours: Effects on Technological, Nutritional, and Consumer Aspects

**DOI:** 10.3390/foods10061213

**Published:** 2021-05-27

**Authors:** Elena Martínez, Rita García-Martínez, Manuel Álvarez-Ortí, Adrián Rabadán, Arturo Pardo-Giménez, José E. Pardo

**Affiliations:** 1 E.T.S.I. Agrónomos y de Montes (UCLM), Campus Universitario s/n, 02071 Albacete, Spain; elena.martinez44@alu.uclm.es (E.M.); rita.garcia@alu.uclm.es (R.G.-M.); manuel.alvarez@uclm.es (M.Á.-O.); adrian.rabadan@uclm.es (A.R.); 2 Centro de Investigación, Experimentación y Servicios del Champiñón (CIES), C/Peñicas s/n, Apdo. 63, 16220-Quintanar del Rey, 160220 Cuenca, Spain; apardo.cies@dipucuenca.es

**Keywords:** food innovation, chia, flax, proximate composition, sesame, poppy

## Abstract

Cookies, which form the largest category of bakery snacks, are considered a good vehicle to introduce nutrients into the diet. In this study, to increase the nutritional value of traditional commercial cookies, wheat flour was substituted with defatted flours made from flax, sesame, chia, and poppy, which are byproducts of the oil extraction industry. The differences in the technological properties, nutritional composition, and consumer acceptance of the reformulated cookies were evaluated. The results show that the wheat cookies used as the control showed a more elastic behavior than the cookies elaborated with defatted seed flours, which showed a greater tendency to crumble. The use of defatted seed flours yielded cookies with a higher content of protein and fiber, and a lower content in carbohydrates than the wheat cookies. Consumer evaluations for the sesame and flax cookies were similar to those for the traditional wheat cookies, with positive assessments on all of the parameters evaluated. On the other hand, the cookies elaborated using chia and poppy flours received the least positive evaluations from consumers. Thus, the use of some defatted seed flours, mainly flax and sesame, is proposed as an interesting alternative to produce health-promoting cookies in order to cover the current demand for gluten-free products.

## 1. Introduction

Cookies are baked products containing three major ingredients: flour, fat, and sugar. They form the largest category of bakery snacks because of their low cost, good taste, texture (crispness), and storability, and are considered an effective vehicle for nutrient supply to consumers. Cookies are usually developed with wheat flour because it forms unique visco-elastic dough when mixed with water, due to the presence of gluten [1]. However, in specific individuals, gluten may create autoimmune reactions. For this reason, the demand for gluten-free products is increasing, leading to a considerable growth in the gluten-free food market [2]. Even among gluten-tolerant individuals, a reduction in the popularity of products with gluten has been observed in recent years [3]. Thus, it would be interesting to substitute wheat for other gluten-free flours that enhance the nutritional quality of the final product [4,5].

However, the nutritional profile of gluten-free bakery products available in the market is often questioned [6,7]. It is thus advisable to replace wheat flour with other flours that provide nutrients and other healthy compounds, and to thus produce cookies that may contribute to the design of a healthy diet. In addition, the healthy properties of snacks are currently a major concern [8,9]. For this purpose, the use of seed defatted flours, which are byproducts of the oil extraction industry, may be appropriate. Several seeds that are commercially available for human consumption as food supplements are considered functional foods because of their beneficial effects on health. This category includes flax, sesame, chia, and poppy seeds. These contain high levels of oil (higher than 20%), which can be extracted by pressure systems, resulting in an oil extraction industry that produces high quality oils rich in polyunsaturated fatty acids. One byproduct of this industry is the press cake, which, once ground, yields the defatted flour. Although these defatted flours may be used for other purposes [10,11], they are generally considered as waste, with no further use. Thus, the incorporation of defatted seed flours into the formulation of cookies may be useful to increase the added value of this byproduct [12].

In addition, defatted seed flours may contain valuable compounds to improve the nutritional properties of cookies. They show higher levels of proteins and total dietary fiber, but a lower content of fat than the seeds [13,14]. However, the remaining fat content has the benefit of seed oils, where polyunsaturated fatty acids are predominant [15]. Additionally, these seeds contain a high proportion of bioactive compounds, such as polyphenolic compounds, with antioxidant properties [16].

In this work, we evaluated the physical, nutritional, and sensory behavior of cookies elaborated with defatted flour from chia, flax, sesame, and poppy seeds, in order to consider the use of this valuable byproduct from the oil industry in the food chain.

## 2. Materials and Methods

### 2.1. Raw Materials

All of the seeds used in this study were acquired in local supermarkets. To prepare the defatted flours, seeds from flax (*Linum usitatissimum*), sesame (*Sesamum indicum*), chia (*Salvia hispanica*), and poppy (*Papaver somniferum*) were subjected to oil extraction with a screw press (Komet Oil Press CA59G, IBG Monforts Oekotec GmbH and Co. KG, Mönchengladbach, Germany) at 49 rpm and 75 °C [17].

### 2.2. Cookies Preparation

The recipe adopted for cookie preparation followed the process proposed by Jan et al. (2018) [4], with slight modifications. First, 25 g brown sugar and 25 g of refined sunflower oil as a fat source were mixed to obtain a creamy mixture. The rest of the ingredients were then added to form the dough, as follows: flour 50 g, sodium bicarbonate 1 g, salt 0.5 g, skimmed powder milk 2.5 g, and water 8 mL. The dough was rolled out with a rolling pin to a uniform thickness of 0.6 cm and cut to form 5 cm diameter round cookies. These were then baked at 200 °C for 10 min. The cookies were subsequently cooled before performing the analysis.

For each type of cookie, the wheat flour as well as defatted flax, sesame, chia, and poppy flours were used separately.

### 2.3. Physical Measurements

We measured the diameter and the height of 10 baked cookies of each type, using a digital caliper. The spreading factor was calculated by dividing the diameter by the height.

The color was measured by reflection in five random points on the surface of the cookies with a Minolta CR-200 colorimeter (Minolta Camera Co. Ltd., Osaka, Japan), using D65 as the illuminant. The tristimulus values were used to calculate the CIELab chromatic coordinates, as follows: L* (brightness), a* (red-green component), and b* (yellow-blue component).

To measure the texture of the cookies, five cookies of each type were cut perpendicularly with a Warner Bratzler blade with a rectangular slot blade at a constant velocity of 2 mm/s, using a TA-XT Plus texture analyzer (Stable Micro Systems, Godalming, UK). We recorded the maximum force needed to cut the cookie and the deformation before breaking.

### 2.4. Proximate Composition

The main nutritional components of the cookies were measured following Rabadán, et al. [18]. Briefly, the protein content was calculated by multiplying the total nitrogen content, obtained by the Kjeldahl method, by a conversion factor of 6.25. To determine the ash content, the flours were ashed at 550 °C to a constant weight. Crude fat (ether extract) was estimated gravimetrically using the filter bag technique after petroleum ether extraction of the dried sample in an Ankom XT10 extraction system. To determine the content of crude fiber, we applied the Weende technique, adapted to the filter bag technique. This method determines the organic residue remaining after digestion with solutions of sulfuric acid and sodium hydroxide, using an Ankom 220 fiber analyzer. The total carbohydrate content was calculated by subtracting the sum of the crude protein, total fat, water, and ash from the total weight of the flour [19]. The available carbohydrate content (nitrogen-free) was calculated by subtracting the crude fiber from the total carbohydrate content [20]. The energy value of the cookies was estimated from the relative content of the protein (N × 6.25), fat, and carbohydrates, using the Atwater general factors of 4.0, 9.0, and 4.0 kcal g-1 for each component, respectively. The proximate analysis was performed in triplicate for each cookie formulation.

### 2.5. Consumer Preferences

To measure consumers’ preferences for the cookies, 106 participants were selected among staff and students at the University of Castilla-La Mancha. Only regular consumers of cookies were involved in the study. With that purpose in mind, only individuals who reported having eaten cookies at least once in the last month were selected for the study. Consumers were asked to score on an 11-point scale how much they liked the recipe, from least (0, I do not like it at all) to most (10, I like it very much). Each consumer spent about 15 min evaluating the cookies. The considered parameters were color, odor, taste, appearance, texture (crunchiness), and overall acceptability of the cookie.

### 2.6. Statistical Analysis

Statistical differences were estimated using an ANOVA test at a 5% level (*p* ≤ 0.05) of significance. All of the statistical analyses were carried out using the SPSS program, release 23.0 for Windows.

## 3. Results and Discussion

### 3.1. Physical Parameters

The spread factor, measured by dividing the diameter of the cookies by their height, is an important parameter to estimate the behavior of the dough during baking. A higher spread factor and larger diameter are considered as crucial quality characteristics for cookies [21]. Although it has been proposed that the gluten, sugar, or fiber content may influence the spread factor [22], in this case, the behavior of cookies was unclear. The spread factor varied from 8.97 in the flax cookies to 11.10 in the poppy cookies, while the cookies made with wheat flour, with gluten, showed intermediate values (Table 1). Significant differences were found in the values between the cookies made with sesame and poppy seed flour. It has been proposed that gluten forms a web during the baking of cookies, which increases the viscosity and stops the flow of cookie dough, leading to lower cookie diameters [23]. However, the diameter of the wheat cookies showed no significant differences with those obtained in the rest of the formulations. This could be explained by the existence of other proteins that may also affect the viscosity of the dough. Regarding the cookies made with defatted seed flours, no clear correlations between spread factor and the rest of nutritional parameters measured were found. Regarding the diameter, the sesame cookies showed significant differences compared with the chia and flax cookies, which showed the lowest values.

**Figure 1:**
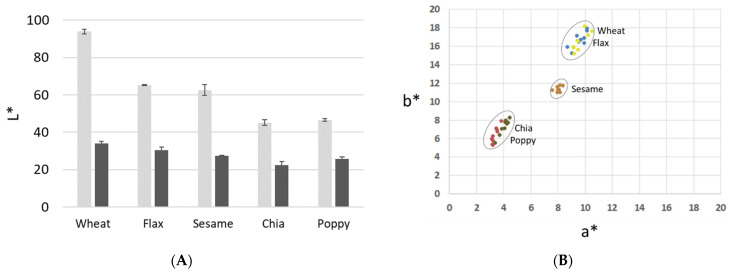
(**A**) Values of brightness (L*) of flour (light grey bars) and cookies (dark grey bars). (**B**) Values of CIELab color coordinates a* (red-green) and b* (yellow-blue) of the cookies elaborated with the different defatted seed flours.

Color was measured according to CIE L*a*b* parameters, where L* represents lightness, a* the value in the red-green axis, and b* the value in the yellow-blue axis. The use of different ingredients has significant effects on the color of the cookies [24]. Surface color is considered an important indicator of the degree of baking, and may play an important role in consumer acceptance. The expected color for cookies is golden brown for the surface and creamish white for the crumb [25]. However, in this case, the color of the cookies was greatly affected by the color of the flour. Chia and poppy flours were darker, resulting in cookie colors with lower L* values. Although baking tends to decrease the lightness of cookies, reducing the difference between them, it is still possible to appreciate significant differences in this parameter (Figure 1A). When the color of the cookies was measured, only those elaborated with flax seed defatted flour showed a similar color to the ones elaborated with wheat flour (Figure 1B). The cookies made from chia and poppy seed flours formed another group with lower values of a* and b*, while the cookies made with sesame flour showed intermediate values. Similar results have been observed when other gluten-free flours are used to make cookies [26]. The lower values of L*, a*, and b* in the cookies elaborated with defatted seed flour indicate less attractive colors for consumers [25]; although, in the case of flax and sesame, the differences with the wheat cookies were smaller.

**Figure 2:**
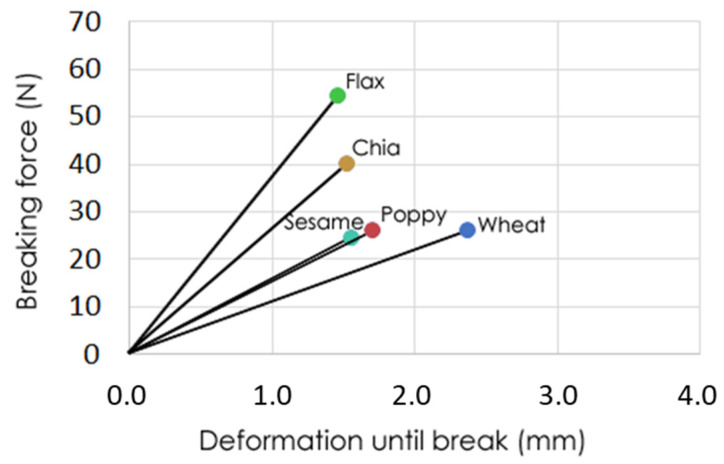
Maximum force and deformation until breaking for cookies elaborated with defatted seed flours. Points are the average value of five repetitions.

Regarding texture, we recorded the maximum force needed to break the cookies and the deformation until the cookies broke (Figure 2). Different patterns were observed for this parameter. The wheat cookies, used as the control, showed a more elastic behavior, represented by the lower slope in Figure 2. This means that the cookies were deformed to a greater degree when force was applied until breaking occurred. The other cookies elaborated with defatted seed flours showed a more fragile behavior, as the deformation was lower when the force was applied. Regarding the maximum force needed to break the cookies, those elaborated with flax flour were the hardest (53.72 ± 11.01 N), followed by chia cookies (39.42 ± 11.42 N), leading to crispier cookies. The cookies elaborated with defatted flours from sesame and poppy showed a similar breaking force to wheat cookies, although deformation until breaking was lower, indicating a more fragile behavior.

### 3.2. Proximate Composition

Table 2 shows the proximate composition of the cookies elaborated with the different seed flours. Humidity was low in all of the cookies, and was lower than 2% in all cases. The reduced values of humidity are adequate to ensure a long shelf-life, making the cookies a long-life food if adequately packaged.

One of the main advantages of seed defatted flours is their high content of proteins [14], which is an interesting source to enhance the nutritional characteristics of baked products. The cookies elaborated with defatted seed flours showed a higher protein content than the wheat cookies. The highest protein content was observed in poppy cookies (17.13%), although no significant differences in flax, sesame, and chia cookies were revealed. In all of the cases, the cookies yielded more than twice the protein content of wheat cookies. Previous studies have reported the addition of defatted sesame seed as a useful addition for increasing the protein content of cookies [27]. Although the main use of these seeds is as oil, because of the elevated proportion of polyunsaturated fatty acids, the benefits of their protein fraction in the defatted flours has also been evaluated [28].

In addition, the fiber content was also higher in all of the cookies elaborated with seed defatted flours. Fiber intake remains low in Western societies, despite the health benefits attributed to it, which are related to metabolic parameters, microbiome composition, and metabolite production [29]. Thus, the fortification of foods with fiber is a major area of interest in the food industry, and is an opportunity for food reformulation [30]. Fiber is an important component of the studied seeds as it represents, for example, 24.65% of the total composition in poppy seeds [31], 27.30% in flax seeds [32], and 34.4% in chia seeds [32]. In this sense, the cookies elaborated with defatted chia flour showed the highest fiber content (12.41%), followed by poppy, sesame, and flax. All of the reformulated cookies showed much higher values than those reported for the wheat cookies (Table 2).

Another important parameter in the proximate analysis is fat content. In this sense, seed flour cookies showed an increase in fat content due to the fat remaining in the flour after oil extraction. When pressure systems are used to extract oil, generally about 15–20% of fat remains in the defatted flour, depending on the oil extraction method [33]. In any event, seed oils show positive characteristics as they are rich in polyunsaturated fatty acids, and this oil can also be considered a healthy source of fat [31]. They are especially rich in α-linolenic acid, a fatty acid the body cannot synthesize and that is the biological precursor to eicosapentaenoic acid and docosahexaenoic acid [34]. Sesame cookies were notable for their fat content (14.11%), because of the lower yield in the oil extraction process and the consequent higher fat content in the defatted flour.

The energy values were similar in all of the cookies, except those made with sesame, due to their higher fat content. The higher protein and fat content in seed flour cookies was counteracted by a lower total carbohydrate content. To reduce the energy value of the sesame cookies, the oil extraction process should be optimized to obtain flour with a reduced fat content [33].

Regarding these data, defatted flours from seeds represent an interesting ingredient to fortify cookies from a nutritional point of view, as they increase the protein and fiber content and reduce carbohydrates. The proximate analysis results suggest that all of the reformulated cookies showed a better nutritional quality than the traditional wheat cookies [35].

### 3.3. Consumer Evaluation

**Figure 3:**
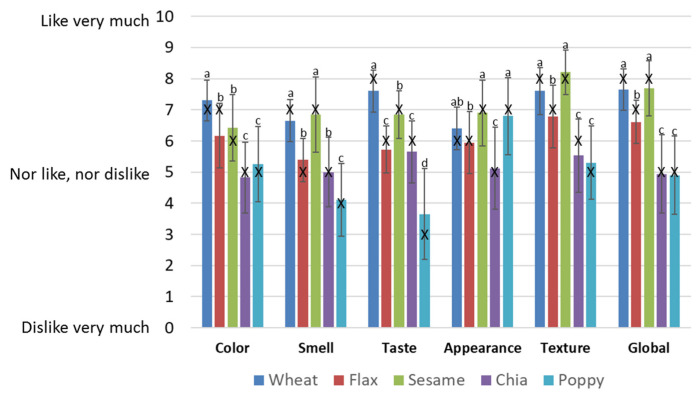
Sensory properties of cookies elaborated with defatted seed flours. Different letters in the same column indicate significant differences (*p* < 0.05).

Regarding the consumer evaluation of cookies, each participant was asked to indicate their preferences on several parameters, namely: color, odor, taste, appearance, texture, and overall acceptance. The data are shown in Figure 3. The color of the cookies made with chia and poppy seed flours was darker than the rest of cookies [24]. As previously reported, consumers prefer golden brown cookies [25], and, consequently, these dark cookies were scored lower by our consumers. On the other hand, the cookies made with flax and sesame flours had positive evaluations, although there were significant differences with the scores obtained by the wheat cookies. Drawing on previous studies, in order to obtain similar values for color in reformulated cookies to those obtained for traditional cookies, traditional flours should only be partially replaced [25]. A significant proportion of consumers are reluctant to try novel foods that differ, in color, for example, to those they usually eat [36].

Regarding odor, again the chia and poppy cookies scored the lowest. In this case, poppy cookies showed values lower than 5, indicating that the odor of these cookies was unpleasant for consumers. Being an odor that consumers are unused to, they consider it a negative attribute. A similar behavior was found when taste was considered, with the poppy cookies scoring lowest, with values below 5. The rest of cookies showed values over 5, indicating that consumers liked them. The sesame cookies showed a median value of 7, the highest of the seed defatted flour cookies, although the highest scores were obtained by the wheat cookies used as the controls. The high values reported for sesame compared with the other seeds could be attributed to the higher fat content. Higher fat content tends to increase consumer preference for various food products, compared with the low-fat versions [37,38].

Regarding texture, cookies made with chia and poppy flours showed a high tendency to crumble, again obtaining a low consumer evaluation. The other three cookies (wheat, flax, and sesame) showed similar crunchy characteristics, leading to higher scores from the consumers, with no significant differences. The global acceptability of sesame cookies showed no significant differences from those of wheat, meaning that consumers could easily change from traditional wheat cookies to 100% defatted flour sesame cookies. Previously, other studies analyzing the overall acceptability of traditional and partially reformulated sesame cookies have reported a similar acceptability in both types [27].

Flax flour cookies also showed positive evaluations in all of the categories tested. On the other hand, the cookies made with chia and poppy defatted flour showed the lowest values regarding sensory evaluation. In this case, other ingredients should be included in the cookie recipe, for example other fat sources [38], to provide better sensory properties that fit consumers’ preferences. These flours could also be mixed, in a low percentage, with wheat flour or other flours to improve acceptance [39].

## 4. Conclusions

Here, cookies were made with seed defatted flour (flax, sesame, chia, and poppy), a byproduct of the oil extraction industry. From a physical point of view, all the cookies made showed a similar behavior when baked, with no clear differences regarding spread factor. The color of the cookies was greatly influenced by the color of the flour, resulting in darker cookies when chia and poppy flours were used. Regarding texture, seed defatted flour cookies showed a higher tendency to crumble, while wheat cookies had a more elastic behavior.

The use of flax, sesame, chia, and poppy defatted flours improves the nutritional properties of cookies, with an increase in protein, fiber, and fat content, but with a decrease in total carbohydrates. From a sensory point of view, the cookies elaborated with flax and sesame defatted flours showed similar values to those of the wheat cookies used as a control on all of the parameters evaluated (color, odor, taste, texture, appearance, and global acceptability). Chia and poppy cookies showed lower values, especially poppy cookies, which yielded values lower than 5 for odor and taste.

All these data suggest that seed defatted flours are an interesting ingredient for improving the nutritional characteristics of cookies, although in the case of chia and poppy, other ingredients, mainly other fats, should be included to improve sensory attributes.

## Figures and Tables

**Table 1:** Mean values for size parameters (diameter and height) and spread factor for all of the cookies.

	Diameter (cm)	Height (cm)	Spread Factor
Wheat	5.34 ± 0.27 ^ab^	0.57 ± 0.03 ^ab^	9.20 ± 0.13 ^b^
Flax	5.22 ± 0.34 ^b^	0.59 ± 0.02 ^a^	8.97 ± 0.97 ^b^
Sesame	5.62 ± 0.22 ^a^	0.54 ± 0.01 ^abc^	10.20 ± 0.16 ^ab^
Chia	5.11 ± 0.21 ^b^	0.51 ± 0.02 ^bc^	9.78 ± 0.37 ^ab^
Poppy	5.31 ± 0.21 ^ab^	0.49 ± 0.06 ^c^	11.10 ± 1.38 ^a^

Numbers are means of multiple measurements. Different letters in the same column indicate significant differences (*p* < 0.05).

**Table 2:** Proximate composition of cookies elaborated with different defatted seed flours.

	Wheat	Flax	Sesame	Chia	Poppy
Humidity (%)	1.6	1.0	1.4	0.8	1.8
Nitrogen (%)	0.96	2.47	2.68	2.55	2.74
Protein (%)	6.00	15.44	16.75	15.94	17.13
Ashes (%)	1.34	3.38	4.27	4.13	6.04
Fiber (%)	0.75	4.03	2.79	12.41	6.96
Fat (%)	4.29	5.93	14.11	5.22	7.07
Total carbohydrates (%)	88.37	75.25	64.87	74.11	69.77
Available carbohydrates (%)	87.62	71.22	62.08	62.30	62.81
Energy value (kcal/100 g)	416	416	453	410	411

Numbers are means of three independent measurements.

## Data Availability

Data available on request.
